# Studying Effects of Gold Nanoparticle on Dose Enhancement in Megavoltage Radiation

**Published:** 2015-12-01

**Authors:** M. Khadem Abolfazli, S. R. Mahdavi, Gh. Ataei

**Affiliations:** 1 Master of Nuclear Physics, Babol University of Medical Science, Paramedical School, Babol, Iran; 2 Assistant Professor of Medical Physics, Iran university of medical science, Tehran, Iran; 3 Master of Biophysics, Babol University of Medical Science, Paramedical School, Babol, Iran

**Keywords:** MCNPX Code, Megavoltage Radiotherapy, Gold Nanoparticle, Dose Enhancement

## Abstract

**Background:**

Gold nanoparticles are emerging as promising agents for cancer therapy and are being investigated as drug carriers, photothermal agents, contrast agents and radiosensitisers.

**Objective:**

The aim of this study is to understand characteristics of secondary electrons generated from interaction of gold nanoparticles GNPs with x-rays as a function of nanoparticle size and beam energy and thereby further understanding of GNP-enhanced radiotherapy.

**Methods:**

Effective range, defection angle, dose deposition, energy, and interaction processes of electrons produced from the interaction of x-rays with a GNP were calculated by Monte Carlo simulations. The MCNPX code was used to simulate and track electrons generated from 30 and 50 nm diameter GNP when it is irradiated with a cobalt-60 and 6MV photon and electron beam in water.

**Results:**

When a GNP was present, depending on beam types used, secondary electron production increased by 10- to 2000-fold compared to absence of a GNP.

**Conclusion:**

GNPs with larger diameters also contributed to more doses.

## Introduction

Nanoparticles have been extensively investigated in diagnostic radiology and radiation therapy. Radio sensitization observed by several authors point out gold nanoparticles (GNP) as the best candidates to be used for flagships of Radionanotherapy. It is well known that dose enhancement can be achieved by introducing a material of high atomic number into a medium such as water when irradiated by kVp x-rays[1, 2]. This effect, which is mainly due to large increase interaction cross-section of photoelectric effect with decreasing energy range, has been extensively studied for cancer radiotherapy[3-8]. Various groups have investigated the possibility of using injected gold nanoparticle (GNPs) for dose enhancement in radiotherapy[9-12]. The dosimetric suitability of GNP application in radiation therapy is one relevant aspect that can justify its applicability in clinical environments.

Increased electron generation by interaction of x-ray photons with GNPs is the main cause of dose increase in GNP-enhanced radiotherapy. Therefore, to understand the role of GNP in enhancing dose, we investigated through Monte Carlo (MC) simulations when nanoparticles were replaced with water. We also investigated the proportion of energy that is self-absorbed by GNP compared to what escapes from nanoparticles. The aim of this study is to understand the characteristics of secondary electrons generated from the interaction of gold nanoparticles GNPs with x-rays as a function of nanoparticle size and beam energy and thereby further understanding of GNP-enhanced radiotherapy. Dose deposition, energy and interaction processes of electrons produced from the interaction of x-rays with a GNP were calculated by Monte Carlo simulations. MCNPX code was used to simulate electrons generated from 30 and 50 nm diameters GNP when it is irradiated with a cobalt-60 and 6MV photon in water. 

## Materials and Methods

### Monte Carlo Simulation Using MCNPX

MC simulations of a GNP irradiated by x-rays in MV energy ranges were carried out using MCNPX code. MCNPX is an MC code that provides a set of physics processes for transport and interactions of particles with matter. Generally, photon and electron transport quantities such as energy deposition were determined by codes based on condensed history. In this study, we used MCNPX code, which gives support for interactions in heterogeneous medium. MCNPX includes routines that allow for the precise definition of complex particle transport volumes and analysis of results. Release 2.4 was used. It is recognized that there is difficulty on this scale to verify the calculated results from microdosimetry experimentally. Nevertheless, it is worthwile for studies such as this one to exist in order to gain physical insights into the phenomenon of radiation dose enhancement due to GNPs. Our findings aim to further the understanding of results from published biological studies regarding GNP-enhanced radiotherapy.

### Simulation Geometry and Setup 

**Figure 1:**
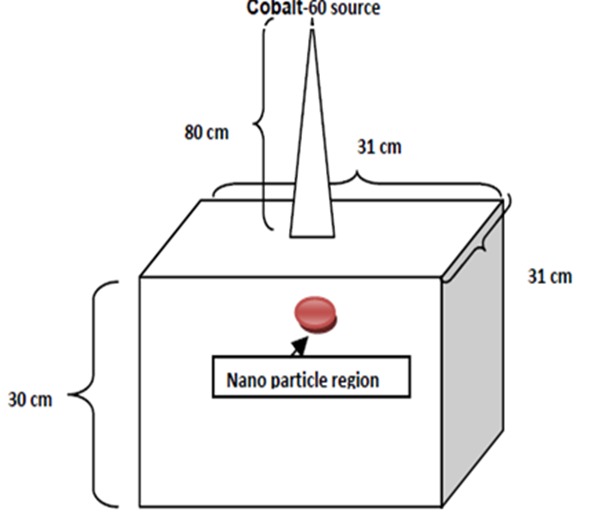
Schematic Diagrams of Monte Carlo Simulation Geometry

Simulation geometry is shown in Figure 1. A spherical GNP, either 30 or 50 nm in diameter, was centered inside a cubic volume consisting of water.

**Figure 2:**
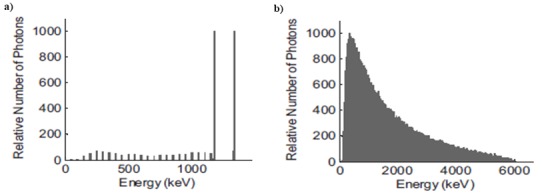
Energy Spectra of Beams Used to Irradiate Nanoparticles for Monte Carlo Simulation. Beams are(a) Cobalt-60 (b) 6 MV.

Nanoparticle was irradiated by 2 polyenergetic photon sources, namely; cobalt-60 and 6 MV. They are shown in Figure 2, normalized to a maximum value of 1000.

All photon paths from the source were parallel to the central axis. The number of primary photons used in each simulation was 250×10^6^. Calculation was performed in a single run without splitting histories. The production threshold, or the energy below which no secondary particle are generated, was set to the minimum of 250ev. Error metric were based on the square root of the number of entries per histogram bin. Simulation time ranged from 20 to 65 h depending on the energy of photon beam. To calculate absorbed dose and energy, deposition of each electron step was recorded. Dose deposited within GNP was adjusted for the density of gold. 

## Results

**Figure 3:**
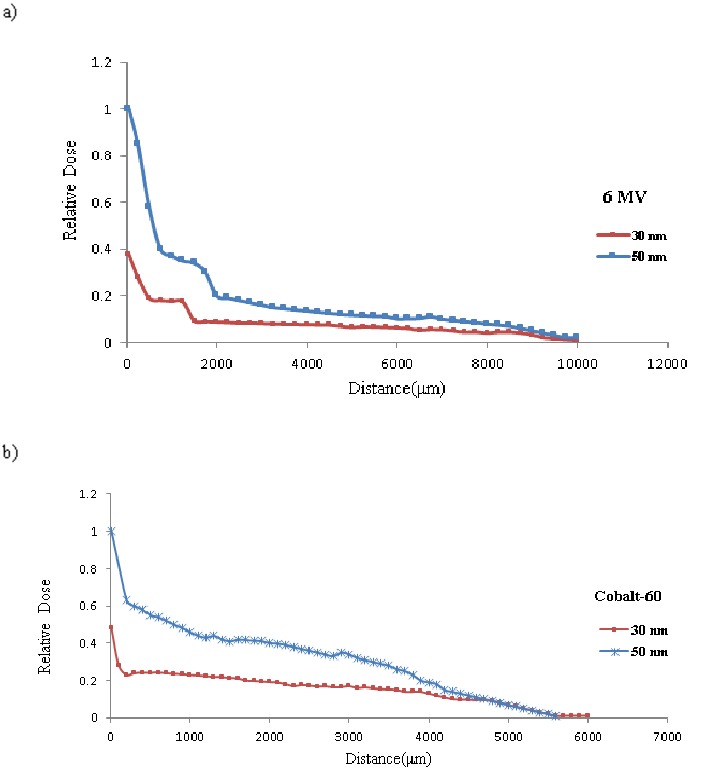
Absorb Dose in Water as Function of Distance away from the Center of Gold Nanoparticle. Data for Beams (a) 6 MV and (b) Cobalt-60.

Figure 3 shows radial dose distribution of a GNP irradiated by photon beams of cobalt-60 and 6 MV, normalized by the maximum dose of each plot. Data show the fall-off of absorbed dose from the center of GNP, excluding the volume within nanoparticles.

The mean energy of electrons as it is injected from the GNP, as well as total relative dose, are summarized in Table 1 for different beams and nanoparticle sizes. Results for water substituted into the GNP volume are also shown for comparison. Note that in this table, a greater uncertainty is associated with the values corresponding to simulations higher energy beams and where GNP is replaced with water due to lower interaction probabilities.

**Table 1:** Results are compared to the case when gold is absent (substituted by water). The calculated dose in this table excludes deposition within nanoparticles volume.

**Features**	**Nanoparticle diameter(nm)**	**Cobalt-60**		**6 MV**	
		**Gold**	**Water**	**Gold**	**Water**
Mean electron Energy(Kev)	30	420 ±10	680 ± 60	370 ± 9	1200 ± 100
	50	454± 9	720 ± 40	386 ± 7	1150 ± 70
Total relative dose outside nanoparticles	30	4.31 ± 0.01×10^-2^	1.36 ± 0.01×10^-2^	3.00 ± 0.01×10^-3^	1.17 ± 0.01×10^-3^
	50	1.005 ± 0.002×10^-1^	2.93 ±0.01×10^-2^	1.088 ± 0.002×10^-2^	1.50 ±0.01×10^-3^

## Conclusions

We have carried out MC simulations to investigate the radial dose distribution, energy, and interaction processes of electrons produced by photon beams’ interaction with a nanoparticle as a function of beam energy and nanoparticle size. The purpose of this study is to gain mechanistic insights to GNP interaction with x-rays in order to account for the dose enhancement observed in gold nanoparticle-enhanced radiotherapy. MCNPX code was used to simulate electron emission from 30 and 50 nm diameter nanoparticles irradiated with parallel photon beams of cobalt-60 and 6 MV. 
